# Discordance in genotypic and phenotypic drug susceptibility results: time to reconsider critical concentration of rifampicin

**DOI:** 10.1128/spectrum.02236-24

**Published:** 2025-02-04

**Authors:** Arti Shrivas, Sarman Singh, Jitendra Singh, Prem Shankar, Payal Soni, Syed Beenish Rufai, Anand Maurya, Shashank Purwar

**Affiliations:** 1 Department of Microbiology, All India Institute of Medical Sciences, Bhopal, India; 2 Aarupadai Veedu Medical College, Vinayaka Mission’s Research Foundation, Pondicherry, India; 3 Translational Medicine Centre, All India Institute of Medical Sciences, Bhopal, India; ICON plc, London, United Kingdom

**Keywords:** tuberculosis, rifampicin, critical concentration, mutations, minimum inhibitory concentration

## Abstract

The objective of this study is to correlate *rpoB* mutations found on the new-generation sequencing (NGS) in *Mycobacterium tuberculosis* (MTB) isolates with minimum inhibitory concentrations (MICs) to the rifampicin (RIF). We assessed the minimum inhibitory concentrations for 151 archived clinical MTB isolates that were determined phenotypically susceptible to RIF (101; 66.89%), and the remaining 50 (50; 33.11%) were resistant to RIF by BACTEC MGIT SIRE DST. MIC values were determined using the colorimetric redox indicator (Resazurin/REMA) method, and results were correlated with *rpoB* gene mutations associated with rifampicin resistance found. Comparing the MIC and critical concentration, we found that 15 of these 101 (14.85%) isolates were misclassified by MGIT-960 as sensitive at standard critical concentration (1.0 µg/mL) though these were found to have low-level RIF resistance by CRI assay (MIC 0.50 to 1.0µg/mL) and NGS. We found that all 15 isolates contained non-synonymous mutations, the commonest being the *Ile572Phe* (7, 46.66%), followed by *Leu533Pro* (3, 20.0%), *His526Leu* (2, 13.33%), *His526Asn+Ile572* Phe (1), *Asp516Tyr* (1), and *Leu533Pro+Pro564* Arg (1). These mutations are reported to confer low-level RIF resistance. But we did not find any mutation at MIC ≤0.25 µg/mL. We found that a significant number of MTB isolates have phenotypic and genotypic discordance. Taking 1.0 µg/mL of rifampicin as a critical concentration, isolates from approximately 15% of patients are misidentified as susceptible to rifampicin, even when these strains carry low-level drug resistance-conferring mutations and have the potential to develop clinical multidrug-resistant tuberculosis.

**IMPORTANCE:**

Tuberculosis (TB) remains a leading cause of morbidity and mortality worldwide, killing millions every year. The emergence of multidrug-resistant and extensively drug-resistant TB forms poses a challenge to the TB control programs. In the past few decades, several molecular tests for rapid detection and drug resistance determination have been developed. But these can miss the genetic mutations that confer low-level resistance to rifampicin (RIF), a critical constituent for treating drug-susceptible TB. On the other hand, for the phenotypic methods, a cutoff value is fixed, known as critical concentration (CC). The current WHO-endorsed CC for rifampicin is 1.0 μg/mL in liquid culture for confirmation of drug resistance; because of that in this system too, low-level RIF resistance may not be correctly identified. Therefore, it is important that either the CC for phenotypic methods is lowered or the specific mutations are included in the molecular tests. This study provides important insights in that direction.

## INTRODUCTION

*Mycobacterium tuberculosis* (MTB) is the causative agent of tuberculosis (TB). Drug-resistant TB has become a major hurdle in the treatment and control of TB around the globe and threatens the progress made so far by the TB elimination programs (1). Patients infected with strains resistant to isoniazid (INH) and rifampicin (RIF), the two most effective first-line anti-TB drugs called multidrug-resistant (MDR) TB, are practically refractory to standard first-line treatment (2). The past decade has seen the increment of multidrug-resistant tuberculosis (MDR-TB) around the globe. The number of primary MDR-TB remained relatively decreased from 3.90% in 2015 to 3.10% in 2020 but started increasing and reported 10.8 million cases in 2023. This sudden increase after the COVID-19 epidemic indicated that after a lull in reporting during COVID-19, the number of reported cases has increased mainly due to restoration of resources (1, 3). India notified 2.42 million MDR cases in the year 2022, an increase of 13.0% as compared to 2021, and reported a total number of 63,801 MDR cases (1, 4).

Rifampicin is an important constituent of the treatment for drug-susceptible tuberculosis. This drug acts against actively growing and slowly metabolizing (nongrowing) bacilli (5). However, prior to the start of MDR-TB treatment, it is necessary to carry out rapid drug susceptibility testing to avoid the misuse of ineffective drugs. In accordance with WHO guidelines, the detection of MDR-TB requires testing for drug resistance using rapid molecular tests, culture methods, or sequencing technologies (2, 6, 7). However, conventional culture-based drug susceptibility testing (DST) also known as phenotypic methods is considered to be the “gold standard” for determining MTB drug resistance at least for most major drugs (8). Even though currently with the availability of whole genome sequence, the latter is considered to be the gold standard, because it provides information not only for the rifampicin resistance–determining region (RRDR) but the entire rpoB gene; most of the commercially available molecular tests such as GeneXpert MTB/RIF and Line Probe Assay/MTBDR*plus* are being progressively applied to detect RR or MDR limiting to only RRDR region.

Resistance to RIF is mainly associated with the mutations in the *rpoB* gene of MTB. Furthermore, more than 95% of MTB clinical isolates that are resistant to RIF have a mutation in an 81 bp “hot-spot” region (HSR) of *rpoB* known as the rifampicin resistance–determining region between codons 507–533 (9). The position of these mutations within the *rpoB* gene is associated with different levels of phenotypic resistance to rifampicin. Some mutations are associated with a high-level resistance to RIF, while others are known to confer only low-level resistance (10). Although the molecular tests have been considered to be highly accurate initially, now some of the clinically relevant mutations conferring resistance are being reported to be missed by these assays because of the location of these mutations outside the targeted region (11, 12).

Similarly, the culture-based methods are also reported to miss clinically relevant RIF resistance in some isolates despite having genetic mutations in *rpoB* region of MTB. The MTB isolates with undisputed mutations in HSR of *rpoB* gene at codons 516, 526, or 531 that cause high-level RIF resistance are readily detected by phenotypic DST methods (13). However, there are frequent discordant results between phenotypic and genotypic assays as shown in various studies (14–17).

Low-level resistance to RIF is clinically significant as patients infected with *M. tuberculosis* strains with disputed *rpoB* mutations often fail treatment or relapse (18, 19). Several recent studies target to identify the characteristics of low-level RR-TB patients worldwide to understand the resistance pattern found, search for similar strains, and identify possible implications for treatment recommendations (20–23).

It has been previously reported in many countries that the mutations associated with low-level resistance are not detected by any of the two WHO-endorsed methods, i.e., phenotypic and molecular methods. Very recently, several reports have been published evaluating the clinical performance of the different phenotypic and WHO-endorsed molecular methods, yet there is limited data on the evaluation of RIF critical concentration in relation to mutations within or outside the RRDR (24).

It is felt that by using the currently WHO-notified critical concentration of RIF, we might be missing several cases of potential RIF resistance, and we might be administering ineffective antituberculosis drug regimen to these patients.

Therefore, in the present study, we aimed to see the concordance between the minimum inhibitory concentration (MIC) in phenotypically RIF-sensitive and RIF-resistant isolates and compared the results with genetic mutations in *rpoB* gene.

## MATERIALS AND METHODS

### Study setting

The study was carried out from April 2020 to September 2021. The study was approved by the Institutional Human Ethics Committee of the All India Institute of Medical Sciences, Bhopal (IHECPGRPHD063).

### Clinical isolates

A well-characterized and anonymized 151 MTB culture isolates were randomly selected from the repository maintained in the accredited TB research laboratory at the All India Institute of Medical Sciences, New Delhi, India. The drug susceptibility pattern of these isolates was previously identified by the BACTEC MGIT960 SIRE DST system. After characterization, these isolates were stored in minus 80°C with in-house culture cataloging until further use. These MTB isolates were freshly subcultured for this work on Lowenstein-Jensen (LJ) medium and in MGIT 960 culture tubes before being used for further phenotypic and genotypic characterization.

### Phenotypic DST

Drug susceptibility testing was performed for all the above 151 MTB isolates by using the BACTEC MGIT 960 SIRE Kit method (Becton-Dickinson, USA). A stock solution was prepared by reconstituting the lyophilized drugs by adding 4.0 mL of sterile distilled water to each vial to make a stock solution of streptomycin (83.0 µg/mL), isoniazid (8.30 µg/mL), rifampicin (83.0 µg/mL), and ethambutol (415.0 µg/mL). All four drugs were used at a final concentration of 1.0 µg/mL (STR), 0.1 µg/mL (INH), 1.0 µg/mL (RIF), and 5.0 µg/mL (EMB) in MGIT tube, respectively. Once reconstituted, the drug solutions were used immediately. The remaining antibiotic solutions were stored at −20°C for future use. Preparation of the inoculum as well as inoculation and incubation were performed as per manufacturer instructions (Becton Dickinson BACTEC MGIT 960 system, BD, USA) (24). A susceptible growth control isolate H37Rv (American Type Culture Collection, 27294) was included in all the experiments.

### Determination of minimum inhibitory concentration for rifampicin

The minimum inhibitory concentration of rifampicin was determined for all the 151 well-characterized *M. tuberculosis* laboratory isolates by colorimetric redox indicator assay using resazurin salt as redox indicator. Briefly, 100 µL of 7H9-S broth (Middlebrook 7H9 supplemented with 0.1% Casitone, 0.5% glycerol, and 10% OADC [oleic acid, albumin, dextrose, and catalase]; Becton-Dickinson) was dispensed in each well of a sterile flat-bottom 96-well plate. One hundred microliters of the working drug concentration (rifampicin, 8.0 µL/mL) was added to the wells containing 7H9-S broth, and the drug was then serially diluted twofold to a final dilution (v/v) of 0.0625 µL/mL as per previously published protocol (25).

An inoculum with turbidity of McFarland standard of 1.0 was prepared from 7H9-S broth and diluted (1:20) in 7H9-S broth for test, and 100 µL of inoculum was added to the drug-free and drug-containing wells. A sterile (no mycobacterium) control and a growth control (with mycobacterium) for each isolate were also included. To prevent evaporation during incubation, sterile water was added to all perimeter wells. The plates were incubated at 37°C after being sealed in a plastic zip-locked bag. A working solution of resazurin salt (30 µL of 0.02% concentration) was added to each microwell after 7 days of incubation. After overnight incubation, plates were read for result interpretation. A color change from blue to pink denoted a positive reaction (reduction of resazurin to resorufin) confirming drug resistance and growth of *M. tuberculosis* cells. The MIC was defined as the lowest concentration of drug that prevents any change of the color of the resazurin from blue to pink indicating the complete inhibition of the growth. Here, the interpretation of “complete inhibition” is when there is “no growth of the bacteria in that particular well and beyond further dilutions in that row”. The wells showing a purple color were considered only partial inhibition of MTB growth (26). The isolates which showed MIC value equal to or higher than 0.50 µg/mL (break point) were considered resistant or uninhibited. For test interpretation, the color was compared to the color present in the growth control well. Confirmation of cell viability was done by inoculating the cells from the particular well on LJ slant. The range of drug concentration we used was 2.0 to 0.0625 µg/mL. Each isolate was tested in duplicates on the same plate.

### DNA extraction

Genomic DNA of twenty-four *M. tuberculosis* isolates was extracted from MTB cultures grown on L-J medium using chloroform isoamyl alcohol method, and the DNA yields were measured by Qubit fluorometry to confirm purity (27).

### Multiplex PCR assay

A multiplex polymerase chain reaction (mPCR) assay was used for the identification and confirmation of *Mycobacterium tuberculosis* Complex (MTBC) before sending the DNA for targeted new-generation sequencing (NGS). The extracted DNA was identified with genus and species-specific primers that are selected to amplify the regions of heat shock protein-65 (hsp-65), early secretory antigenic target-6 (esat-6), and internally transcribed sequence (*its*) to detect Mycobacterium genus, *M. tuberculosis* complex species, and *M. avium* complex (MAC) species, respectively (28). PCR products were electrophoresed on 1.8% agarose gel and visualized under UV *trans* illuminator; results were interpreted by *M. tuberculosis*-specific bands at 441 bp (*hsp-65*) and 320 bp (*esat-6*) and 126 bp (*its*).

### Primer designing and sequencing

The PCR primer sets targeting inside and outside RRDR region of *rpoB* gene, *M. tuberculosis* H37Rv (GenBank accession no: NC_000962), were designed by Primer3software (http://primer3.ut.ee/) and custom-synthesized from Eurofins Genomics India Pvt. Ltd. Bangalore, India. The sequencing primers used were forward: 5′-ATG ACC ACC CAG GAC GTG-3′ and reverse: 5′-ACA CGA TCT CGT CGC TAA CC-3′. A PCR assay with sequencing primers was also performed as a quality control check on DNA extracts to assess the sequencing of *rpoB* gene and quantitation of *Mycobacterium tuberculosis* DNA.

After performing the MIC on all 151 isolates, 20 isolates that were detected sensitive by MGIT DST showed discordance in REMA, and 4 isolates that were detected resistant by MGIT DST and REMA that also showed the same results in MIC analysis were further tested. These 24 isolates that showed low-level RIF resistance (MIC ranging from ≥0.25 to <1.0 µg/mL) were sequence-confirmed by new-generation sequencing. Furthermore, to determine the level of phenotypic resistance with mutations associated with *rpoB* gene in *Mycobacterium tuberculosis,* the PCR products were purified by using QIAquick PCR purification kit protocol. The purified amplicons were commercially target sequenced from Anuvanshiki (OPC) Pvt Ltd., New Delhi, using automated sequencer (Applied Biosystem).

### Sequence analysis

The obtained sequences were processed and analyzed through MEGA X for mutational study. Mutations in *rpoB* gene sequences were compared with wild-type sequences of H37Rv strain of *M. tuberculosis* using ClustalW tool (http://www.ebi.ac.uk/Tools/msa/ clustalw2) and again performed multiple sequence alignments (MSA) using ClustalW tool.

## RESULTS

A total of 151 well-characterized MTB culture isolates were randomly selected for this study. Among these, 50 (33.11%) were resistant to rifampicin, singly or in combination with other drugs, and 101 (66.89%) isolates were phenotypically sensitive to RIF. The drug susceptibility pattern of these isolates is given in Table 1.

**TABLE 1:** Phenotypic drug susceptibility patterns of the MTB culture isolates (*N* = 151)^*a*^

S. No.	RIF susceptibility status	Sensitive/resistance to drugs	No. (%)
1.	RIF-sensitive isolates (*n* = 101; 66.88%)	STR+INH+RIF+EMB Sensitive	70 (69.3%)
		INH Mono-Resistant	20 (19.8%)
		STR Mono-Resistant	3 (3%)
		EMB Mono-Resistant	2 (2%)
		INH+EMB Resistance	5 (4.9%)
		INH+STR Resistant	1 (1%)
2.	RIF-resistant isolates (*n* = 50; 33.11%)	RIF+INH Resistant	20 (40%)
		RIF+INH+STR Resistant	14 (28%)
		RIF+INH+EMB Resistant	9 (18%)
		STR+RIF+INH+EMB Resistant	6 (12%)
		RIF Mono-Resistant	1 (2%)

^*a*^ RIF, rifampicin; INH, isoniazid; STR, streptomycin; EMB, ethambutol.

### Minimum inhibitory concentration

**Fig 1:**
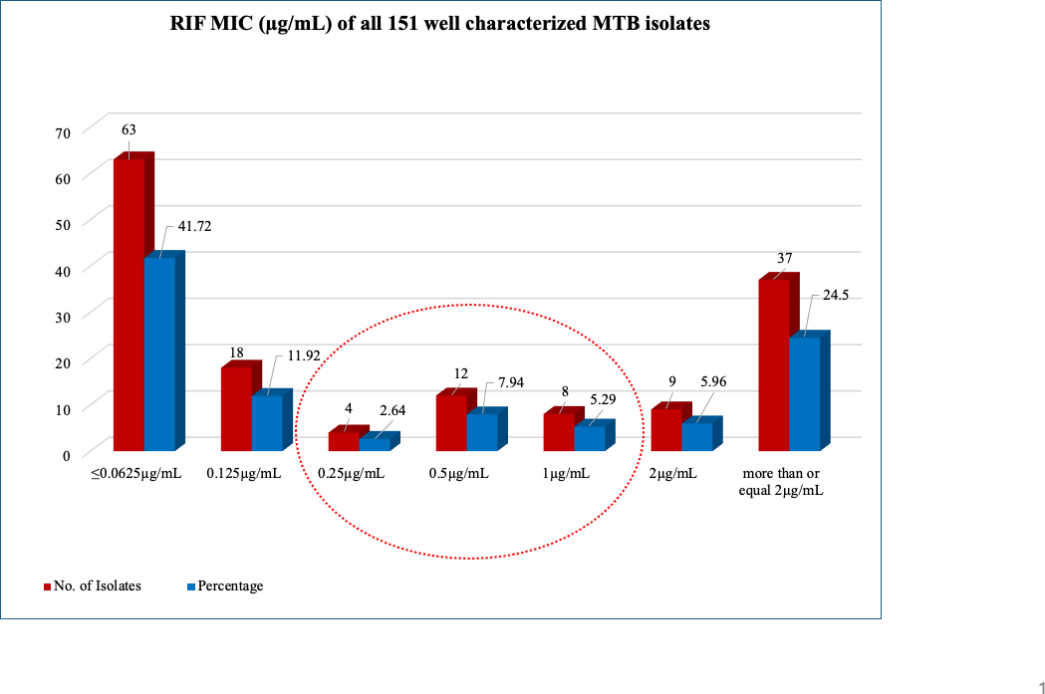
Results of the rifampicin MIC (in µg/mL) for all 151 well-characterized MTB isolates.

Out of the 151 isolates tested, 18 (11.92%) had MIC of 0.125 µg/mL, and 63 (41.72%) had MIC of ≤0.0625 µg/mL. These were considered sensitive to RIF by CRI and MGIT-960 system as well (at critical concentration 1.0 µg/mL). While 4 (2.64%) isolates had MIC of 0.25 µg/mL, 12 (7.94%) had MIC of 0.5 µg/mL, and 08 (5.29%) had MIC of 1.0 µg/mL. These were identified as low-level RIF resistant (29). The 46 (30.46%) isolates, having MIC of ≥2 µg/mL, were considered high-level rifampicin-resistant isolates (Fig. 1). The 24 isolates that were identified as having low-level RIF resistance (MIC ranging from ≥0.25 to ≤1.0 µg/mL) by CRI assay were sequenced, as highlighted in Fig. 1.

### RRDR gene sequencing results

**Fig 2:**
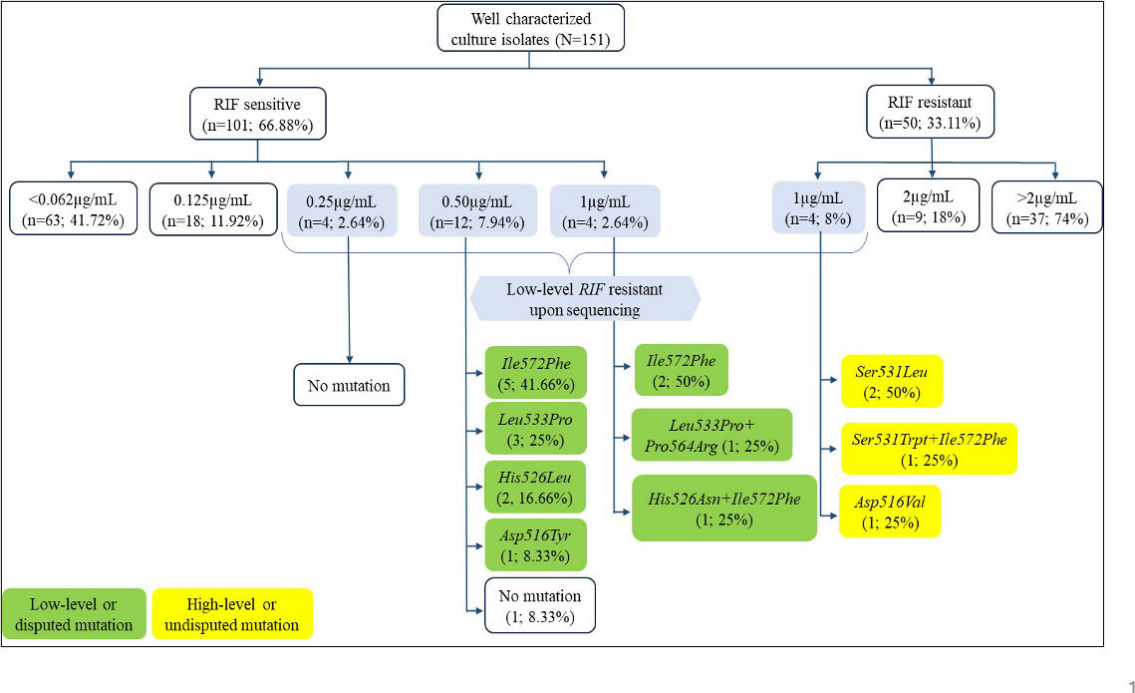
Distribution of the *rpoB* gene mutations confirmed by sequencing in the studied *M. tuberculosis* isolates (*n* = 24).

The sequencing results of the 24 isolates revealed that mutations in *rpoB* gene, within or outside RRDR region, were detected in 19 isolates (Fig. 2). The remaining three isolates revealed double mutations that included both inside and outside the RRDR [TCG-TGG (*Ser531Trp*) + ATC TTC (*Ile572Phe*)1; CAC-AAC (*His526Asn*) + ATC TTC (*Ile572Phe*)1; CTG-CCG (*Leu533Pro*) + CCT-CGT(*Pro564Arg*)1]. We did not find any mutation in the four isolates having MIC value of 0.25 µg/mL.

We found that the majority of these isolates exhibiting low-level phenotypical RIF resistance were carrying disputed *rpoB* mutations. These included *His526Leu*, 2; His*526Asn*, 1; *Asp516Tyr*, 1; *Leu533Pro*, 4; and *Ile572Phe*, 9, as shown in Table 2.

**TABLE 2:** Distribution of mutations and MIC of the isolates (*n* = 24)^*a*^

S. No.	Mutations		RIF MIC (µg/mL)	No. of isolates
	Nucleotide change	Amino acid change		
1	ND	ND (4)	0.25	4
2				
3				
4				
5	ATC-TTC	*lle572Phe* (5)	0.5	12
6				
7				
8				
9				
10	CAC-CTC	*His526Leu* (2)		
11				
12	GAC-TAC	*Asp516Tyr* (1)		
13	CTG-CCG	*Leu533Pro (3*)		
14				
15				
16	ND	ND (1)		
17	TCG-TTG	*Ser531Leu* (2)	1	8
18				
19	TCG-TGG, ATC-TTC	*Ser531Trp, Ile572Phe* (1)		
20	GAC-GTC	*Asp516val* (1)		
21	ATC-TTC	*lle572Phe* (2)		
22				
23	CAC-AAC, ATC-TTC	*His526Asn, Ile572Phe* (1)		
24	CTG-CCG, CCT-CGT	*Leu533Pro, Pro564Arg* (1)		
Total				24

^*a*^ ND, not detected.

## DISCUSSION

Rifampicin resistance is considered a surrogate marker of multidrug-resistant tuberculosis. Successful treatment of MDR-TB is based on a timely and correct diagnosis and the drug susceptibility test, which provides evidence for selecting an effective second-line drug regimen. In the past 15 years, several molecular diagnostic methods, which allow the rapid detection of drug resistance in MTB directly from specimens, have been developed, overcoming the limitations of conventional phenotypic methods. Also, a number of these commercial molecular assays have been endorsed by WHO, which include GenoType MTBDR*plus* (Hain LifeScience, Germany), GeneXpert MTB/RIF (Cepheid, USA), and Truenat MTB (Molbio Diagnostics Pvt. Ltd., India). These assays detect RIF resistance-conferring genetic mutations in the rifampicin resistance–determining region. However, the biggest limitation of these tests is that they miss the genetic mutations that are outside the target (RRDR) region and often considered as are disputed to confer the clinical drug resistance (11). Moreover, the molecular tests are unable to determine the level of phenotypic resistance. On the other hand, the WHO-endorsed liquid culture-based phenotypic DST, which remains the gold standard, is also not able to detect all clinically relevant resistant cases.

Using the phenotypic methods, to level an isolate as sensitive or resistant, the isolate has to be tested *in vitro* under the drug challenge. Critical concentration (CC), which is defined as the minimum concentration of the antituberculosis drug in the culture medium that is able to inhibit 99.0% growth of *M. tuberculosis*, has a crucial consideration. This concentration keeps on changing depending on the baseline resistance to a particular drug at the particular period, in that group of population. The WHO recommended 1.0 µg/mL as critical concentration for RIF resistance in BACTEC Mycobacterial Growth Indicator Tube 960 (MGIT) DST method, which is endorsed as a gold standard for phenotypic drug susceptibility testing and initiating the treatment according to these cutoff values. The manufacturers (Becton-Dickinson, USA) have also standardized the system to flash these isolates as drug resistant or drug susceptible, using this critical concentration (30).

Recent studies have also shown that liquid culture systems such as MGIT- 960 using a critical concentration of 1.0 µg/mL often fail to detect strains exhibiting low-level (e.g., MIC of <1.0 µg/mL) resistance to RIF (29). These low-level RIF-resistant strains with MICs below the critical concentration mostly contain specific mutations, within the hot-spot region of the rpoB gene (HSR-*rpoB*).

Strains with high-level resistance will have clearly elevated RIF MIC (≥16.0 µg/mL), and phenotypically these are correctly identified as resistant in MGIT. Similarly, strains having low-level resistance with RIF MIC between 0.25 and 1.0µg/mL will be identified phenotypically susceptible at in MGIT. Several of these isolates might have clinical resistance (30). Torrea et al. (29) reported that by using the lower level of MIC, a high number of isolates could be leveled as resistant. In their study when the critical concentration was reduced to 0.50 µg/mL, they detected 10% additional cases, and when the critical concentration was further reduced to 0.25 µg/mL, an additional 25.70% cases could be detected (26). Shea et al. (30) also reported similar results where they reported that 18.9% of strains were RIF susceptible in MGIT at 1.0 µg/mL but had low-level RIF resistance by MIC testing (29). Others have also reported similar observations (11).

In our study, 15 isolates that were detected as sensitive by MGIT at standard critical concentration (1.0 µg/mL) but found to exhibit low-level RIF resistance by CRI assay were sequence-confirmed for mutations that reported to confer low-level resistance by NGS based upon the presence or absence of known *rpoB* mutations. Among them, we observed that all the isolates contained non-synonymous mutations, the commonest being the *Ile572Phe*, followed by *Leu533Pro*, *His526Leu*, *His526Asn + Ile572* Phe, *Asp516Tyr*, and *Leu533Pro + Pro564* Arg (Fig. 2).

**Fig 3:**
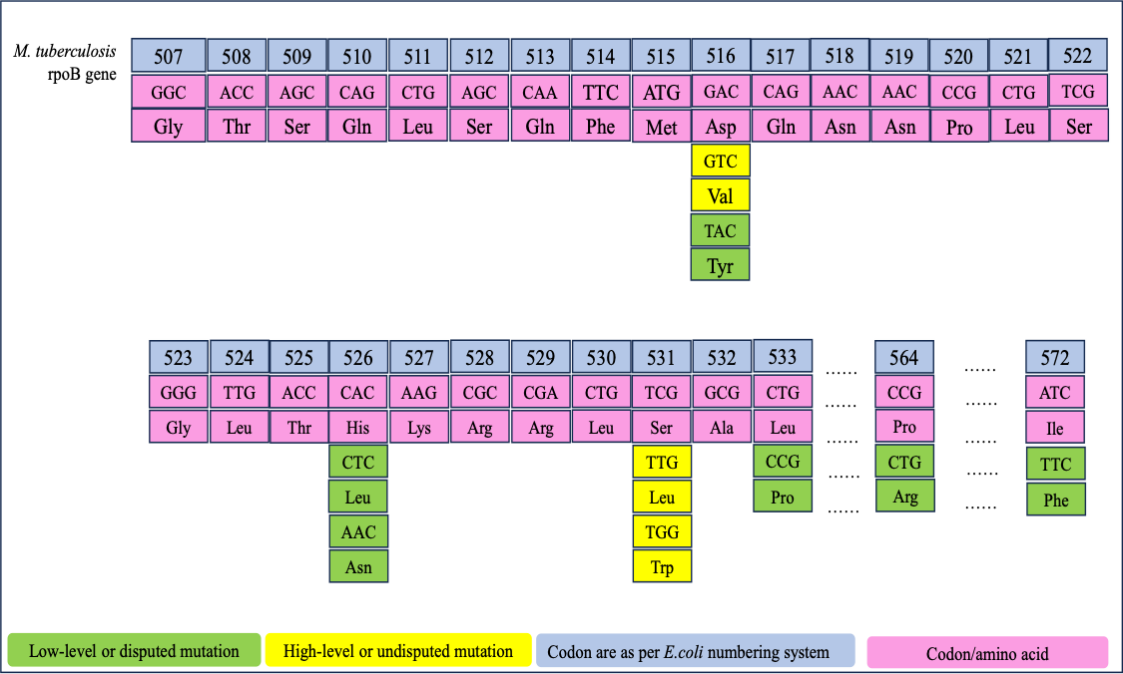
Schematic representation of codon positions of *rpoB* gene at which mutations were found in our study. Diagramatic represenation of *M. tuberculosis rpoB* gene mutations with codon numbering based on *E. coli* system. Low-level mutations like *Asp516Tyr* and high-level mutations like *Ser531Leu* are highlighted, along with corresponding amino acid changes.

These are reported to be disputed mutations exhibiting low-level RIF resistance. The codon positions of *rpoB* gene at which mutations were found in our study are shown in Fig. 3. However, there is no consensus on how much reduction in the presently WHO-recommended critical concentration of 1.0 µg/mL can be done or if should it be changed at all. The WHO Technical Expert Group in 2020 deliberated on this issue and agreed that the critical concentration of 0.50 µg/mL would be idealistic, but it could not verify with CLSI if there is sufficient evidence to recommend lowering the critical concentration of RIF from 1.0 to 0.50 µg/mL in MGIT culture-based drug susceptibility testing (3). The WHO also considers that if the critical concentration of RIF is reduced from 1.0 to 0.50 µg/mL in MGIT culture-based drug susceptibility testing, the number of RIF-resistant cases, which is also the surrogate marker of MDR-TB, will increase exponentially. It may not be feasible for the national TB control programs to handle such a large number of resistant cases, which are hitherto considered to be susceptible using the currently recommended critical concentration of 1.0 µg/mL.

The current guidelines of WHO are mostly reliant on molecular detection of resistance conferring mutations, because the molecular tests are rapid and less cumbersome and can be done directly on the clinical samples without even culturing the pathogen. However, these molecular tests including the GeneXpert MTB/RIF and LPA have a limited scope of detecting the genetic mutations in a specific (hotspot) area of *rpoB* gene. Any mutation outside the hotspot (e.g., *Ile572Phe*) or on the extreme ends of RRDR region will be missed, and these patients will be treated with standard regimen of drug-susceptible TB cases. While actually, these patients need to be treated with an MDR-TB regimen (11, 15, 26).

The distribution of these mutations seen in our study is in line with previous studies (11, 29), but the frequencies of various mutations were different. In our study, we found that *Ile572Phe* mutation was commonest, while Torrea et al. (29) found that *Asp516Tyr* was the commonest, and Rigouts et al. (11) found that *His526Leu* was the commonest mutation when using the MIC of 0.50 µg/mL (29). In a recent report (31), out of 308 isolates, 227 isolates were found to be RIF-susceptible by culture-based DST, but 8 (3.52%) of them were identified as resistant by MTBDR*plus*. On WGS, they found that two isolates had *Leu533Pro* mutations, another two had *Asp516Tyr*, and one each had *His526Leu*, *Leu511Pro*, and *His526Asn* mutations. The MICs of these isolates ranged from 0.125 to 1.0 μg/mL. Testing of RIF MICs was done on MGIT 960 system. We did not find any of these mutations at MIC ≤0.25 µg/mL. In our study, 20% of isolates were found to have *Leu533Pro* mutation at MIC of 0.5 µg/mL which is the highest frequency reported so far. Rando-Segura et al. (31), from Angola reported a frequency of 12.50%, Torrea et al. (26) from Belgium reported 11.11%, and Salaam-Dreyer et al. from South Africa reported the frequency of 8% (32) only. While Berrada et al. from the USA reported a very low frequency of 2.3% only (33). At MIC of 0.50 µg/mL, we also found that *His526Leu* mutation was present in 13.33% of isolates. The frequency of this mutation was much higher in our isolates as compared to other studies, where the frequency ranged from as low as 2.4% to 12.5% (26, 31–34). None of the mutations reported in our study are listed in the WHO catalogue (34). The WHO catalog list mutations in rpoB were found to be associated with RIF resistance. The WHO has grouped these mutations into five groups. The grouping is made to understand the accuracy of these mutations with the clinical and phenotypic resistance. To further minimize the confusion, these mutations are classified according to the expert rule that any RRDR mutation, with the exception of synonymous mutations, should be assumed to confer RIF resistance. This expert rule was first introduced by WHO in 2018 and reaffirmed in 2021 (34). However, the mutations reported here do not fall into the expert rule and hence not included in the catalogue. This further provides an opportunity to include these mutations in more studies and analyze their potential in clinical use. The high frequency in our isolates could be justified by high the TB burden in India, and ours was the recent study, indicating faster mutation rates in the isolates circulating in India.

In our study, the frequency of mutation *Asp516Tyr*, *His526Asn + Ile572* Phe, and *533Pro + Pro564* Arg was low at MIC values between 0.5 and 1 µg/mL. Others have also reported low frequency of these mutations, except Andres et al. from Germany who reported a prevalence of 25% (35). These minor and non-consequential changes in various reports could be due to methods they used for MIC determination; nevertheless, all these studies including our study have included WHO-endorsed break points and critical concentrations in standard tests of that time.

At MIC of 1.0 µg/mL, we found that *Ile572Phe* mutation was commonest (13.33%). This frequency falls within the range reported by other workers (30, 32). The *His526Leu* mutation was found in our isolates at this drug concentration, but others have reported its frequency up to 12.5% (31). We could detect *Leu533Pro* mutation in 6.6% of our isolates, while Ho et al. from Australia reported a prevalence of 20% (18), and Wang et al. from China reported frequency of this mutation in 3.3% of isolates (18, 36).

Our study also showed novel findings of dual mutations as mentioned above. Such dual mutations have not been reported by anyone so far. As such, not many studies have been done on this aspect to correlate the MIC and mutations but even very few on the low-level resistance conferring mutations. There is only one study from India (37). Of the 84 RIF-resistant isolates, they found 16.66% isolates having disputed mutations, at MIC of 0.25 to 0.5 µg/mL, *Asp516Tyr* in 1.2%, and *His526Asn* in 2.38%, respectively. Therefore, ours become the unique study in which we have compared the frequency of various mutations often considered to be disputed or low resistance conferring with the low MIC in the MGIT culture. Our study is not only the first of its kind from India but also from the entire Southeast Asia to the best of our knowledge and literature search until the writing of this manuscript.

Several recent studies have argued in favor of lowering the critical concentration. Yip et al. (38) from Hong Kong proposed that strains carrying *Leu511Pro*, *Leu533Pro*, and *His526Leu* mutations represented almost 22% (19/85) of all the mutations compared to less than 10% among all phenotypically RIF-resistant strains of previous years, indicating approximately 12% phenotypically RIF-susceptible isolates (38). They also mentioned that the frequency of these mutations may vary from region to region. This further poses a question if the frequency of these mutations also varies in various genotypes (39).

From our study and from others’ work, it is evident that lowering of the critical concentration in phenotypic methods and inclusion of the abovementioned mutations and their probes in currently used molecular methods can detect several such cases which are hitherto considered as drug susceptible, and treatment with primary anti-TB drugs is initiated. Our data show that a significant number of isolates carry low-level RIF resistance and the patient carrying these strains are falsely leveled as susceptible and thereby treated with primary anti-TB drugs. Many of these patients might later manifest as MDR cases. This will be worthwhile to conduct nationwide research to find these mutations and follow these patients who carry these so-called low-level or disputed mutations, to see what percentage of these patients actually get cured using the primary antitubercular disease. This evidence is necessary before advising lowering the critical concentration and including these mutations in the currently used molecular tests because this might pose a huge human resource and economic burden on the national TB elimination/control programs.

We are aware that impromptu lowering of the critical concentration in DST might pose a huge human resource and economic burden on the national TB elimination/control programs. Hence, we propose that it will be worthwhile to conduct nationwide research to detect these mutations in RIF-susceptible patients and follow these patients to see how many of these patients get cured with primary anti-TB drugs. We hypothesize that a significant number of patients carrying low-level drug resistance mutations will become clinically drug resistant.

### Conclusion

While correlating the MIC values with genetic mutations within the HSR-*rpoB* gene region of *M. tuberculosis*, using current standard critical concentration of 1.0 µg/mL for rifampicin, we observed that in a significant number of clinical isolates, there is a significant amount of disagreement. This translates to the fact that a significant number of patients are put on an ineffective antituberculosis drug regime.

Spontaneous reduction of critical concentration in drug susceptibility testing may impose significant human resource and economic challenges on national tuberculosis elimination and control programs. Consequently, we propose that it would be beneficial to undertake research aimed at identifying low-level drug resistance mutations through innovative diagnostic techniques. Additionally, it would be important to monitor these patients to determine the percentage that achieves successful outcomes with first-line anti-TB medications, as well as those requiring second-line treatment regimens. We hypothesize that a considerable proportion of individuals harboring low-level drug resistance mutations may ultimately develop clinically significant drug resistance.
